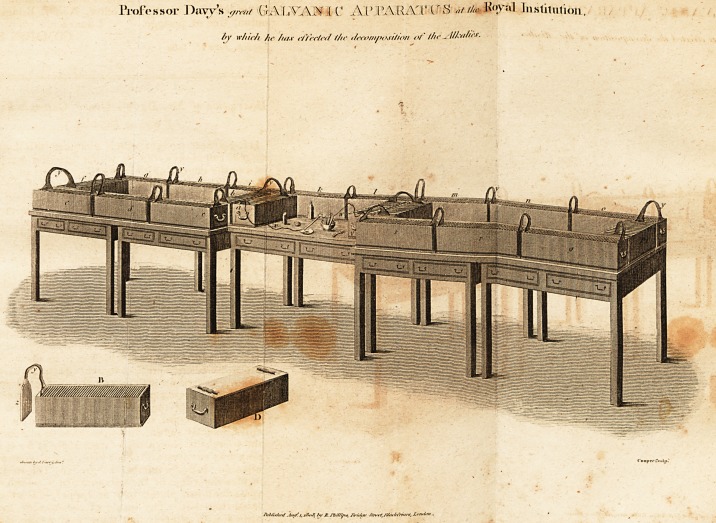# Description of Mr. Davy's Grand Galvanic Battery

**Published:** 1808-09

**Authors:** 


### *Description of Mr.* Davy's *Grand Galvanic Battery.*

( With an Engraving. )

THE galvanic troughs which compose this battery are arranged round five tables, which are too particularly shewn in the engraving to require any letters of reference. The trough *a* is the first of the positive end of the battery, the wire *x* coming from it, conveys the effect of all the battery to the place where the experiments are made, which is in the clear space upon the centre table; the next trough is *b*, connected with *a* by means of a piece of thin sheet lead *y*; the troughs then follow round the tables in the orders of the letters of reference, *c d e f g h i k l m n o p q r s t w*, each being connected with the adjacent one by a piece of lead *y*, until we arrive at *t* and *o*, where two troughs, corresponding with *a* and *b*, are placed in the mid dle of the tables; the trough *v* is the first of the negative, and has a copper wire *w* proceeding from it, which is wrapped round the end of a small probe of platina; the positive wire *x* is connected with a small platina dish or saucer; the piece of potash which is to be decomposed, is placed in this dish, and a sufficient quantity of rectified naphtha is poured on it to defend the product from the atmosphere; and being touched by the platina probe, it receives the shock of the whole battery, and very quickly the metalloid begins to appear upon the dish in small globules, exactly similar in appearance to mercury. One of the troughs is shewn on a larger scale at *b*; it is a ma hogany box twenty-two inches long, and five and a half within side: it has a number of grooves cut in its sides, to receive the edges of the galvanic plates, which are each composed of two plates, one of copper, the other zinc, soldered together. The inside of the box or trough, is varnished with cement, that there may be no communication from one of the cells, formed between the plates and the next; in putting the plates together they must be arranged

### Professor Davy's *great* GALVANIC APPARATUS *at the* Royal Institution,

*by which he has effected the decomposition of the Alkalies.*

*drawn by J. Farey, Junr.*

Cooper sculp.

*Published Augt. 1, 1808, by R. Phillips, Bridge Street, Blackfriars, London.*

arranged, to be alternately zinc and copper; that is, the zinc side of one plate opposite the copper side of the next. The troughs have each of them two pieces of glass tube stuck upon the bottom of the trough, as shewn at *d*, to support it; by this means the troughs are insulated, so that the galvanic fluid cannot escape to the earth when the batterry is in use. The cells between the plates are filled with sulphuric acid, diluted with water; and in order to connect the effect of two or more troughs together, two plates similar to *z* are joined together by a thin piece of sheet lead, soldered to both; one of the plates *z* is copper, the other zinc. One of the plates is put into the last cell of each trough, and the lead conveys the electric fluid from one trough to the other; as the lead is so easily bent, the trough can be set down without any particular regard to position, and the lead bent to reach into them both. There are twenty troughs with twenty-five plates in each, making five hundred plates; the superficies of each plate exposed to the action of the acid, contains thirty-six square inches.

## Figures and Tables

**Figure f1:**